# Supplementary material for: Food-washing monkeys recognize the law of diminishing returns
Source: eLife. 2025 May 22;13:RP98520. doi: 10.7554/eLife.98520 (PMC12097787; doi:10.7554/eLife.98520)
Supplement: Supplementary file 1. [file elife-98520-supp1.docx]

Summarized fixed effects for the food brushing GLMM (n = 575 events by animals with known rank) as an analysis of deviance table (Type II Wald Chi Square Tests).

| **Fixed Effect** | **𝛸^2^** | **Degrees of Freedom** | **p (one sided)** |
| --- | --- | --- | --- |
| Grit treatment | 194.67 | 2 | **p < 0.0001** |
| Ordinal rank * grit treatment | 2.45 | 2 | 0.29 |
| Sex | 0.24 | 1 | 0.62 |
| Ordinal rank | 0.21 | 1 | 0.65 |
