# Supplementary material for: Food-washing monkeys recognize the law of diminishing returns
Source: eLife. 2025 May 22;13:RP98520. doi: 10.7554/eLife.98520 (PMC12097787; doi:10.7554/eLife.98520)
Supplement: Supplementary file 2. [file elife-98520-supp2.docx]

Summarized fixed effects for the food washing GLMM (n = 362 events by animals with known rank) as an analysis of deviance table (Type II Wald Chi Square Tests) for the model that included both quadratic and linear ordinal rank terms.

| **Fixed Effect** | **𝛸^2^** | **Degrees of Freedom** | **p (one sided)** |
| --- | --- | --- | --- |
| Grit treatment | 69.71 | 2 | **p < 0.0001** |
| Ordinal rank^2^ * grit treatment | 19.29 | 2 | **p < 0.0001** |
| Ordinal rank | 0.15 | 1 | 0.70 |
| Ordinal rank^2^ | 0.14 | 1 | 0.70 |
| Sex | 0.01 | 1 | 0.92 |
