# Supplementary material for: Food-washing monkeys recognize the law of diminishing returns
Source: eLife. 2025 May 22;13:RP98520. doi: 10.7554/eLife.98520 (PMC12097787; doi:10.7554/eLife.98520)
Supplement: Supplementary file 3. [file elife-98520-supp3.docx]

Summarized fixed effects for the food washing GLMM (n = 362 events by animals with known rank) as an analysis of deviance table (Type II Wald Chi Square Tests) for the model that included just a linear ordinal rank term.

| **Fixed Effect** | **𝛸^2^** | **Degrees of Freedom** | **p (one sided)** |
| --- | --- | --- | --- |
| Grit treatment | 66.44 | 2 | **p < 0.0001** |
| Ordinal rank * grit treatment | 18.54 | 2 | **p <0.0001** |
| Ordinal rank | 0.06 | 1 | 0.80 |
| Sex | 0.06 | 1 | 0.81 |
