# Supplementary material for: Food-washing monkeys recognize the law of diminishing returns
Source: eLife. 2025 May 22;13:RP98520. doi: 10.7554/eLife.98520 (PMC12097787; doi:10.7554/eLife.98520)
Supplement: Supplementary file 4. — Modeled as a zero-inflated Poisson (ZIP) (n = 575 observations). [file elife-98520-supp4.docx]

Full model fixed effects and confidence intervals for the food brushing GLMM. Modeled as a zero-inflated Poisson (ZIP) (n = 575 observations).

| **Fixed effect** | **Estimate** | **Std. Error** | **Z value** | **P (two-sided)** | **Lower CI** | **Upper CI** |
| --- | --- | --- | --- | --- | --- | --- |
| Ordinal rank | 0.002 | 0.01 | 0.16 | 0.87 | -0.03 | 0.03 |
| Grit treatment, low | -7.80 | 2.42 | -3.22 | **0.00129** | -12.54 | -3.05 |
| Grit treatment, medium | -1.47 | 0.19 | -7.85 | **p <0.0001** | -1.84 | -1.10 |
| Sex, Male | 0.10 | 0.21 | 0.49 | 0.62 | -0.30 | 0.51 |
| Ordinal rank* grit treatment, low | 0.11 | 0.16 | 0.68 | 0.50 | -0.20 | 0.42 |
| Ordinal rank*grit treatment, medium | 0.02 | 0.02 | 1.42 | 0.15 | -0.01 | 0.05 |
