# Supplementary material for: Food-washing monkeys recognize the law of diminishing returns
Source: eLife. 2025 May 22;13:RP98520. doi: 10.7554/eLife.98520 (PMC12097787; doi:10.7554/eLife.98520)
Supplement: Supplementary file 6. — Modeled as a zero-inflated Conway-Maxwell Poisson (ZICMP) (n = 362 observations). [file elife-98520-supp6.docx]

Full model fixed effects and confidence intervals for the food washing GLMM fixed effects with only a linear ordinal rank term. Modeled as a zero-inflated Conway-Maxwell Poisson (ZICMP) (n = 362 observations).

| **Fixed effect** | **Estimate** | **Std. Error** | **Z value** | **P (two-sided)** | **Lower CI** | **Upper CI** |
| --- | --- | --- | --- | --- | --- | --- |
| Ordinal rank | -0.11 | 0.24 | -0.44 | 0.66 | -0.57 | 0.36 |
| Grit treatment, low | -5.90 | 1.54 | -3.84 | **0.0001** | -8.92 | -2.89 |
| Grit treatment, medium | -4.62 | 0.98 | -4.72 | **p < 0.0001** | -6.54 | -2.70 |
| Sex, Male | -0.81 | 3.35 | -0.24 | 0.81 | -7.38 | 5.76 |
| Ordinal rank* grit treatment, low | 0.17 | 0.09 | 1.80 | 0.07 | -0.015 | 0.35 |
| Ordinal rank*grit treatment, medium | 0.23 | 0.06 | 4.15 | **p < 0.0001** | 0.12 | 0.35 |
